# Rehabilitative interventions for ischaemic digital ulcers, pain, and hand functioning in systemic sclerosis: a prospective before-after study

**DOI:** 10.1186/s12891-022-05145-8

**Published:** 2022-03-02

**Authors:** Dalila Scaturro, Giuliana Guggino, Pietro Terrana, Fabio Vitagliani, Vincenzo Falco, Daniele Cuntrera, Maria Grazia Benedetti, Antimo Moretti, Giovanni Iolascon, Giulia Letizia Mauro

**Affiliations:** 1 Department of Surgical, Oncological and Stomatological Disciplines, University of Palermo, Palermo, Italy; 2 Rheumatology section, Biomedical department of Internal Medicine, University Hospital “P.Giaccone”, Palermo, Italy; 3 University of Palermo, Palermo, Italy; 4 University of Catania, Catania, Italy; 5 Department of Economics and Statistics, University of Palermo, Palermo, Italy; 6 Department of Economics and Statistics, University of Palermo, Palermo, Italy; 7 IRCCS-Istituto Ortopedico Rizzoli- University of Bologna, Bologna, Italy; 8 Department of Medical and Surgical Specialties and Dentistry, University of Campania “Luigi Vanvitelli”, 80138 Naples, Italy; 9 Department of Surgical, Oncological and Stomatological Disciplines, University of Palermo, Palermo, Italy

**Keywords:** Rehabilitation, Systemic sclerosis, Ultra-sound therapy, Manual therapy, Connective tissue disease

## Abstract

**Background:**

Systemic sclerosis (SSc) is a rare connective tissue disease characterised by immune dysfunction, vasculopathy, cellular inflammation, fibrosis of the skin associated with multiple internal organs involvement. Ischaemic digital ulcers (IDU) of the hands commonly occur in patients with SSc adversely affecting functional independence.

**Purpose:**

Aim of the study is to investigate the effectiveness of a rehabilitation protocol based on the combined use of ultrasound (US) therapy and therapeutic exercise in terms of ulcers healing, pain relief, and hand functioning in patients affected by SSc with IDUs. Moreover, we also investigated the safety of the proposed intervention.

**Study design:**

Prospective before-after study.

**Methods:**

We included 20 patients with IDUs secondary to SSc. All patients were treated with US combined with manual therapy, including McMennel joint manipulation, pompage mobilization technique and connective tissue massage, for 10 sessions. We evaluated softness, dyschromia, pain, and hand mobility using the Pressure Sore Status Tool (PSST), the Numerical Rating Scale (NRS), and the Duruoz Hand Index (DHI) at T0 and at the end of the treatment (T1).

**Results:**

Treatment with US combined with manual therapy significantly reduced ulcers depth, improved ulcers margins, and reduced periwound skin damage (median PSST score 16 at T1, *p*<0.0001). Moreover, significant benefits were reported in terms of pain relief (NRS 3 at T1; *p*<0.0005), and hand function (DHI score 19 at T1; *p*<0.0005). Finally, this approach seems to be safe, without side effects reported at the end of treatment, along with an optimal compliance.

**Conclusion:**

Therapeutic US combined with manual therapy should be used as additional intervention to manage IDUs in SSc patients.

## Background

Systemic sclerosis (SSc) is a rare connective tissue disease characterised by immune dysfunction, vasculopathy, cellular inflammation, fibrosis of the skin associated with multiple internal organs involvement. The extension of the skin involvement enables to classify the disease in a limited cutaneous form (lcSSc), where the affected skin is restricted to the hands, forearms, feet, and face; on the contrary in patients with diffuse cutaneous involvement (dcSSc) the affected skin extends proximal to the elbows and may involve the trunk [1].

In 2017, ACR and EULAR published new criteria for the classification of SSc. Skin thickening of the fingers extending proximal to the metacarpophalangeal joints (MCP) is a sufficient criterion for the diagnosis of SSc. In addition, seven other variables are considered, including skin thickening of the fingers, digital tip ulcers or pitting scars, telangiectasia, abnormal nailfold capillaroscopy, pulmonary arterial hypertension and/or ischaemic digital ulcers (IDU), Raynaud’s phenomenon, and SSc-related autoantibodies (i.e., anticentromere, anti-topoisomerase I, anti-RNA polymerase III) [2].

Ischaemic digital ulcers are chronic painful lesions that occur equally on hands and feet. Hypoxic damage begins with skin thickening on distal phalanges and may develop to dermal and hypodermic necrosis and even to osteolysis.

The progressive nature of the disease leads to important disability that needs proper rehabilitation approach. In particular, hands dysfunction causes marked decline in performing the activities of daily life (ADL). In fact, joint stiffness progresses toward contractures resembling “claw deformities”, with the loss of MCP and proximal interphalangeal joints (PIP) flexion [3].

To date, several pharmacological treatments for IDUs have been proposed, while for rehabilitation approaches (i.e., exercise, manual therapy, and physical modalities) no specific indications have been provided [2, 4, 5].

Aim of the study is to investigate the safety and the effectiveness of a rehabilitation standardized protocol based on the combination of ultrasound (US) therapy and therapeutic exercise to improve ulcer healing, pain, and hand function in patients with IDUs secondary to SSc.

## Materials and methods

### Patients

A prospective before-after monocentric study was conducted between June 2020 and November 2020. Twenty patients were selected and enrolled by the Rheumatology Unit of the "P. Giaccone" Polyclinic in Palermo and sent to the Rehabilitation Unit of the same University Hospital to perform the functional assessment and the rehabilitation treatments.

Inclusion criteria were as follows: diagnosis of systemic sclerosis according to ACR and EULAR criteria for SSc [2], complicated by persistent active ulcers on the fingertips; iloprost suspended for at least three months for poor or no response to the treatment; during the study period patients did not receive any specific medical therapy for IDUs to avoid confounding factors in assessing the effectiveness of the interventions.

Patients with infectious diseases (e.g., HIV, HBV, HCV), acute inflammation, cancer or rheumatoid arthritis were excluded.

Patients were recruited in June and treated within the end of July. We have recorded an average temperature difference between two months of just 3-4°C.

### Intervention

All patients were treated with US (I-Tech medical device certified UT2 CE0476) combined with manual therapy, including McMennel joint manipulation, pompage, and connective tissue massage for a total duration of 100 minutes for each session, for a total of ten consecutive sessions. Dipping technique was applied for therapeutic US with a frequency of 1 MHz, intensity of 1 W/cm^2^, duty cycle of 60% and a duration of 15 minutes per session. The technique involves immersion of the hands in a metal container with a diameter of 90 cm, previously sanitized with an alcoholic solution, containing 4 liters of water at a temperature of 37-37.5°. Furthermore, inside the container there is an emitter handpiece with a radiant area of 5 cm^2^, at a distance of 2 cm from the body surface.

The McMennel manipulation is a rehabilitation technique used to reduce joint stiffness and to improve joint lubrication and pain through the stretching of the capsuloligamentous complex [6]. It was used for 40 minutes.

The connective tissue massage (lasting 30 minutes) aims at preserving the softness and smoothness of the skin by promoting blood circulation, also playing a muscle relaxant role [7].

The mobilization technique of pompage is a type of manual therapy, widely used in the treatment of musculoskeletal disorders, consisting of applying a tension of the fascia, with slow and progressive mobilizations, through a rhythmic and regular movement of traction and release [7]. It is a method that focuses on the treatment of the fascia, has a duration of 15 minutes and it can be divided into three phases: tension; maintenance; and gradual release.

The rehabilitation protocol consisted of 10 sessions performed daily from Monday to Friday for two consecutive weeks.

### Outcomes

The enrolled patients have been evaluated by the following scale: Pressure Sore Status Tool (PSST), numerical rating scale (NRS) and the Duruoz's hand index (DHI). Furthermore, hand-held photographs were carried out by the operator.

The Numerical Rating Scale is a quantitative assessment tool that ask the patients to rate their pain on a defined scale, from 0 to 10, best reflects the intensity of the pain in that specific moment [8].

The Duruoz Hand Index (DHI) is a self-report questionnaire designed to evaluate activity limitations of the hand. It comprises of eighteen items which are scored on a 6-point scale where 0 is “without difficulty” and 5 is “impossible” [9].

The Pressure Sore Status Tool (PSST) scores is an evaluation tool consisting of 13 items that evaluate the characteristics of the wound and surrounding tissue in terms of size, depth, edges, weakening, type of necrotic tissue, amount of necrotic tissue, type of exudate, exudate, skin color of the surrounding wound, peripheral tissue edema, peripheral tissue hardening, granulation tissue, epithelization. Each item is given a score based on a Likert scale of 5 points. The total sum of all scores gives the PSST score. The higher the final score, the more serious the state of the injury considered [10].

The follow-up evaluations were performed at 30 days after the end of the 10 rehabilitative sessions.

The study was approved by the Medical Ethical Committee of the University Hospital of Palermo, Italy (N.6/2020); informed consent was obtained from each patient in conformity with Helsinki Declaration.

### Statistical analysis

Descriptive analysis was performed by median and confidence intervals. Differences of the parameters between the first and the second examination (T0 and T1) were compared using the Mood test on the medians for the ordinal nature of the variables. The Wilcoxon signed-rank test was used to assess the variation of the PSST ordinal score between a pre and a post treatment. All analyses were performed using R software. Values of *p*-value less 0.05 were considered statistically significant.

## Results

The general characteristics of the enrolled patients (14 females and 6 males) are summarized in Table 1.

**Table 1:** Patient’s characteristics at baseline

Characteristics	
Mean Age (years)	61.05
Gender, n (%)	
*Men*	6 (30%)
*Women*	14 (70%)
NRS (mean)	5.55
Ulcer number (mean)	2
Ulcer size	
*< 4 cm*^*2*^	13
*4 – 16 cm*^*2*^	7

All patients completed the proposed treatment with a compliance of 100% and without side-effects at the end of treatment period. Of the 20 patients recruited, only 4 patients were on immunosuppressants/immunomodulators (such as rituximab and Azathioprine) due to concomitant pulmonary complications (e.g., interstitial lung disease). The remaining patients, in addition to skin involvement, had no further manifestations of systemic sclerosis.

Patients showed a significant reduction for NRS pain after treatment. At T0, 50% of patients had a NRS between 5.5 and 7, with median value of 5.5 (moderate), while at T1 50% of them had a NRS between 3 and 5, with median value of 3 (mild) (*p*<0.0005) (Table 2).

**Table 2:** Effectiveness of the intervention on pain, hand functioning and ulcer healing in SSc patients with IDUs

Outcome measures	T0			T1			***p***-value
	Median	25th percentile	75th percentile	Median	25th percentile	75th percentile	
NRS	5.5	5	6.25	3	2	4	**<0.0005**
DHI	25	21.5	37	19	15.75	25.25	**<0.0005**
PSST	23	21	28	16	14.75	17.25	**<0.0005**

**Fig. 1:**
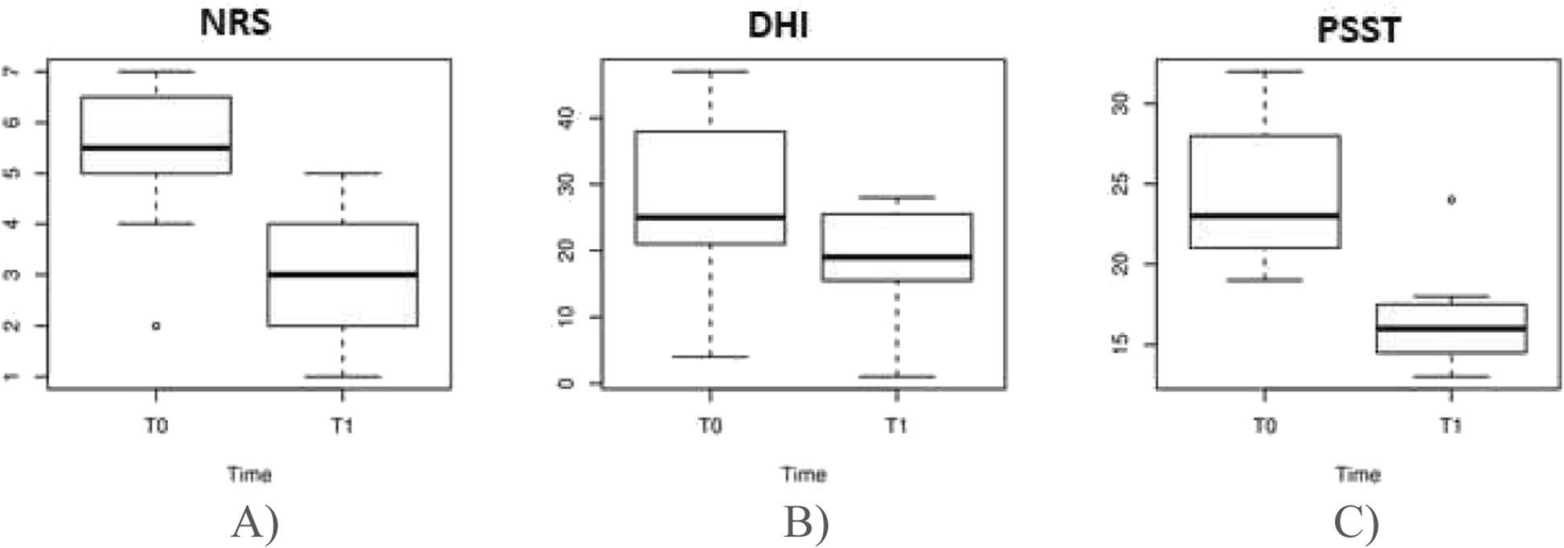
Boxplot of the measurements made at T0 and T1 for NRS (**A**), Duruoz Hand Index. (DHI) (**B**) and Pressure Sore Status Tool (PSST) (**C**)

Duruoz Hand Index and PSST scores also resulted significantly reduced after treatment, respectively from a median value of 25 at T0 to 19 at T1 (*p*<0.0005) for DHI, and from 23 at T0 to 16 at T1 for PSST (*p*<0.0005) (Table 2). Figure 1 shows the boxplots, at T0 and T1, for NRS, DHI, and PSST.

**Fig. 2:**
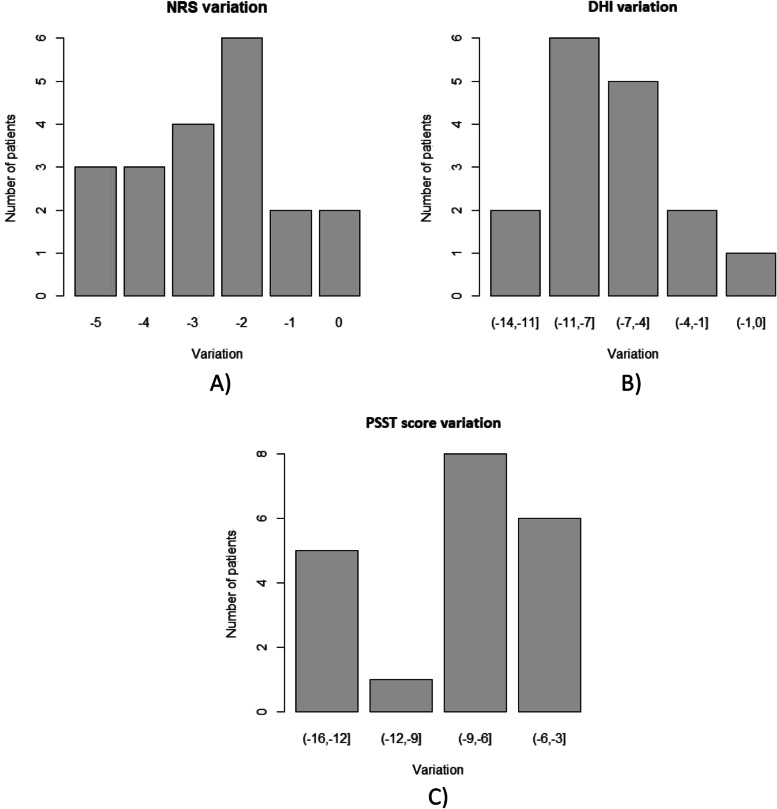
Clinical variations of NRS (**A**), DHI (**B**) and PSST (**C**)

The variations of each outcome measure in our cohort are detailed in Fig. 2. The Fig. 2A shows the changes in patient’s NRS. All patients improved, except for two patients who did not have benefit. Two of them scored 1 point less, while most patients (16 patients) had a 2-point decrease on the NRS. The best benefit recorded was a 5-point decrease, which was obtained by 3 patients. In Fig. 2B the DHI’s changes in patients and their relative frequencies are reported. All patients improved, except one patient. Two patients had a variation between 1 and 3, five patients a variation between 4 and 6, six patients a variation between 7 and 10 and two patients a variation between 11 and 13 points. As far as regards variations of the PSST score (Fig. 2C), an improvement was observed for all patients. Six patients had a variation between 3 and 5, eight patients a variation between 6 and 8, one patient a variation between 9 and 11 and five patients a variation between 12 and 15 points.

More specifically, through the Wilcoxon rank test we highlighted significant changes due to the treatment only in 5 items of the PSST score: size, depth, edge, epithelization, and skin color.

**Fig. 3:**
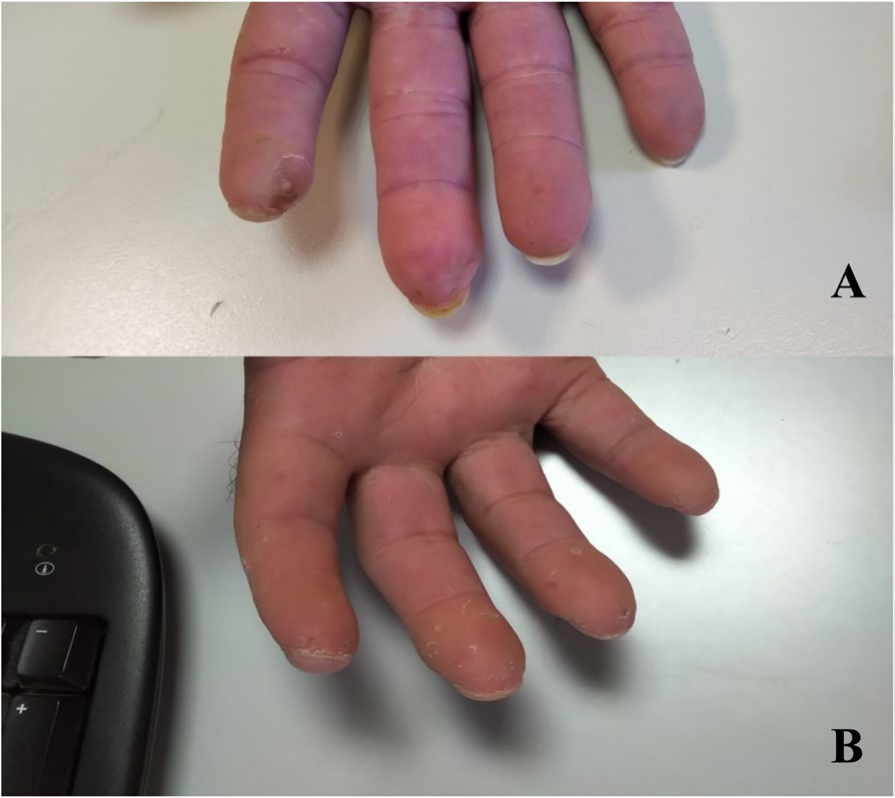
Ischemic hand ulcers before (**A**) and after (**B**) treatment

In all the patients the size of the wound improved or did not worsen. In the item “size” of the PSST, 13 patients had 1 point at both T0 and T1, 7 patients had 2 points at T0 and 1 point at T1 (Δ T1-T0, *p*=0.016). Depth of the wound (item “depth” of the PSST) improved in 11 patients, while no improvement was found in 9 patients: 1 patient had 1 at both T0 and T1, 8 patients had 2 at both T0 and T1, 10 patients had 2 points at T0 and 1 point at T1, 1 patient had 3 points at T0 and 1 point at T1 (Δ T1-T0, *p*=0.001). For the edge of the wound (item “edges” of the PSST), 4 patients had 1 point at both T0 and T1, 12 patients had 2 points at T0 and 1 point at T1, 4 patients had 3 points at T0 and 1 point at T1 (ΔT1-T0, *p*<0.0001). “Epithelialization” item of the PSST improved in 9 patients, while no improvement was found in 11 patients: 3 patients had 1 point at both T0 and T1, 8 patients had 2 points at both T0 and T1, 9 patients had 2 points at T0 and 1 point at T1 (Δ T1-T0, *p*=0.004). Skin color (item “skin color surrounding wound”) improved in 15 patients while no improvement was found in 5 patients: 5 patients had 2 points at both T0 and T1, 15 patients had 2 points at T0 and 1 point at T1(Δ T1-T0, *p*<0.0001). The remaining items did not change significantly. Clinical improvements in IDU are illustrated in Fig. 3.

## Discussion

Ischaemic digital ulcers lead to significant pain and functional limitations of the hands in patients with SSc [11]. Our study investigated the effectiveness of an innovative and easy to perform rehabilitation protocol for the management of patients with IDUs secondary to SSc, including therapeutic US and manual therapy. In the literature there are no similar studies evaluating this intervention strategy in the management of IDUs.

Therapeutic US reduces inflammation of tissues and improves cell proliferation, collagen production, neoangiogenesis and fibrinolysis [12, 13]. Low US frequencies can reduce bacterial counts on wounds through selective destruction of bacterial biofilm [14].

The McMennel method prevents the development of deformity of the claws, resulting in an improvement in hands’ mobility, and improves hand muscles’ extrinsic strength in order to decrease pain and joint stiffness [15].

The mobilization technique of pompage allows reaching the optimal elongation of the collagen fibers that form the fascia, obtaining the recovery of the physiological lengths of this structure, as well as having analgesic effects, and promotes local blood circulation [15]..

Therapeutic US combined with hand rehabilitation using manual therapies (the McMennel method, connective tissue massage, pompage) has proven to be safe in this population, as it is free of side effects, and it might be included among the conservative approaches to SSc patients with IDUs as ancillary intervention to the recommended pharmacological therapies [2]. Furthermore, the painless and short duration of US technique promotes greater compliance [16], as demonstrated by treatment adherence and persistence.

The treatment and prevention of IDUs is an important component in the management of patients with SSc. The European League against Rheumatism (EULAR) guidelines recommend only pharmacological approaches as gold standard for treating active digital ulcers in patients with SSc, such as intravenous iloprost. However, repeated infusions of this drug require patient hospitalisation and are associated with serious adverse effects (i.e., headache, flushing, nausea, vomiting, jaw pain, myalgia) [15]. There are multiple topical treatments proposed for IDUs in the literature [17], although no consensus about this therapeutic option has been reached. The application of topical hydrocolloid and occlusive substances may be useful to protect the affected skin and to prevent the outbreak of further ulcers [17].

Ozone therapy has been shown to be useful in promoting IDUs healing in patients with SSc, through induction of VEGF and release of oxygen stimulating antioxidant enzymes [17].

Lipofilling is a minimally invasive method that can induce improvement or healing in SSc-related IDUs that are resistant to other traditional therapeutic approaches. It is a technique widely used in cosmetic (anti-ageing), surgery, but also for the treatment of post-surgical scars and radiotherapy-induced lesions. The method consists of taking fat tissue from the patient (liposuction) and re-injecting it into a different body region [18].

Debridement of IDUs has shown a reduction in ulcer-related pain and improved healing of the tissues involved, although no standardised protocols are available [19].

Physical modalities have been also proposed to treat IDUs in SSc patients. In this population, application of extracorporeal shock wave therapy (ESWT) has been shown to be well tolerated, repeatable, painless, and effective. It results in improvement of digital ulcers, skin elasticity and general well-being, however beneficial effects tend to reduce over time [20]

Recently, the local administration of a vasodilator (i.e., treprostinil) through iontophoresis has been proposed. This treatment increases the blood flow of the skin in the leg and foot compared to a placebo. Despite some minor local adverse events (e.g., erythema or burns), related to iontophoresis, this procedure has been shown to be safe and effective in the treatment of digital ulcers in patients with systemic sclerosis [21].

Promising results are obtained by using botulinum toxin type A (BTX-A) injections for treating chronic and refractory IDUs. The authors state that treatment with BTX-A is a minimally invasive method that resulted in a significant reduction in perceived pain, with an average duration of effect for 8 months, and a reduction in the intake of vasodilator medicines. Hand function and grip strength also showed significant improvements [22].

Although few studies support rehabilitative interventions for patients with SSc, these approaches are widely used in clinical practice. In agreement with our results, Bongi et al., demonstrated the effectiveness of the combination of connective tissue massage and the McMennel method on pain, joint mobility, and hand function [23]. Horváth et al. also support the effectiveness of physiotherapy in the treatment of IDUs from SSc [24].

The strength of the study is the originality of the proposed treatment, as the combination of therapeutic US and of manual therapy has never been studied in patients with SSc-related IDUs. Moreover, also considering that digital ulcers heal over time even without interventions, our population consisted of patients complaining of IDU persistence for several months.

The main limitations of the study are the sample size, the study design (pre-post study), and the lack of a control group. Another limitation of this study is the lack of a long-term evaluation of the benefits obtained in terms of healing and prevention of ulcers. On the other side, it must be considered the difficulty in finding participants as SSc is a rare disease.. The results obtained might open a future line of research that should be implemented through randomized controlled trials. In the future, we would improve the study design to carry out a more reliable investigation including the comparison of treatment effects with a control group.

## Conclusions

Based on our data, we demonstrated the effectiveness of therapeutic US combined with manual therapy in reducing ulcer depth and margins, improving perilesional skin colour and fullness, and in improving hand function in SSc patients with IDUs.

In conclusion, our data support the use of rehabilitative interventions as additional therapeutic strategy for improving the management of IDUs in patients with SSc.

## Data Availability

data will be available on reasonable request to dalila.scaturro@unipa.it

## Abbreviations

SSc: Systemic sclerosis
IDU: Ischaemic digital ulcers
US: Ultrasound
PSST: Pressure Sore Status Tool
NRS: Numerical Rating Scale
DHI: Duruoz Hand Index
lcSSc: Limited cutaneous form
dcSSc: Diffuse cutaneous involvement
MCP: Metacarpophalangeal joints
ADL: Activities of daily life
PIP: Proximal interphalangeal joints
EULAR: European League against Rheumatism
ESWT: Extracorporeal shock wave therapy
BTX-A: Botulinum toxin type A
